# The Influence of Aging Temperature and Cryogenic Treatment on the Mechanical Properties and Microstructure of Extruded Mg-8Gd-3Y-0.4Zr Alloy

**DOI:** 10.3390/ma18122922

**Published:** 2025-06-19

**Authors:** Haoran Pang, Lunyuan Tang, Xiaojun Wang, Min Ma, Liwei Lu

**Affiliations:** 1 Hunan Rongtuo New Material Research Co., Ltd., Xiangtan 411201, Chinaxjwang@hit.edu.cn (X.W.); mma@hnust.edu.cn (M.M.); 2 Sanya Institute of Hunan University of Science and Technology, Sanya 572024, China; 3 National Key Laboratory of Precision Hot Processing of Metal, Harbin Institute of Technology, Harbin 150001, China

**Keywords:** Mg-8Gd-3Y-0.4Zr alloy, aging treatment, cryogenic treatment, microstructure, hardness

## Abstract

This investigation implemented an integrated aging–cryogenic thermal processing method for extruded Mg-8Gd-3Y-0.4Zr alloy to further improve its performance and broaden its scope of application, employing a characterization approach combining optical microscopy (OM), electron backscatter diffraction (EBSD), X-ray diffraction (XRD), and transmission electron microscopy (TEM). The comprehensive microstructure characterization was systematically correlated with mechanical property evolution to establish structure–property relationships. The results show that aging combined with cryogenic treatment significantly enhances the hardness and improves the microstructure of magnesium alloys. Specimens aged at 210 °C for 20 h followed by one-hour cryogenic treatment exhibited the highest average hardness (113.5 HV), representing a 11.2–25% improvement compared to those aged at lower temperatures. This enhancement can be attributed to the elevated aging temperature promoting substantial precipitation and subsequent growth of second phases such as Mg_3_(Gd,Y), which benefit from sufficient thermal activation energy. The increased density and larger dimensions of these second phases contribute to enhanced hardness through elevated internal stress generation. However, their non-uniform distribution may induce localized stress concentration, consequently reducing hardness uniformity. Notably, specimens subjected solely to 210 °C aging for 20 h showed marginally lower hardness compared to their cryogenically treated counterparts, suggesting that although cryogenic treatment may refine grain structures and introduce dislocation defects to enhance hardness, its concurrent reduction in residual stresses might limit the overall improvement magnitude.

## 1. Introduction

Mg alloys, acknowledged as the lightest metallic structural materials, demonstrate substantial industrial significance and compatibility with sustainable energy initiatives, thus being acclaimed as the “green engineering material of the 21st century” [1]. These alloys present notable advantages, including exceptional specific strength-to-weight ratios, remarkable stiffness, superior thermal conductivity, and favorable biocompatibility, making them viable candidates for critical applications across aerospace systems, transportation engineering, 3C electronics manufacturing, and biomedical device development [2,3,4]. Specific examples include electronic product housings and automotive seat frames. Nevertheless, their widespread implementation is constrained by intrinsic limitations such as low absolute mechanical strength and room-temperature plastic formability [5]. To improve the properties of magnesium alloys, researchers have added rare-earth elements into magnesium alloys. Among them, the Mg-Gd-Y system has attracted much attention due to its low density and good mechanical properties [6]. And, based on this, processing techniques are adopted to further improve the performance.

Extrusion is a conventional processing method for magnesium alloys, featuring low cost, high efficiency, and the ability to effectively refine the grain structure. However, the improvement in mechanical properties achieved solely through extrusion is insufficient for demanding applications. As a conventional heat treatment, aging treatment has been extensively implemented in metallic material engineering to enhance mechanical properties through precipitation strengthening of second phases [7,8]. Xu et al. [9] conducted a solution treatment at 788 K for 4 h followed by an aging treatment at 498 K for 12 h. The material exhibited significantly enhanced mechanical properties due to the formation of densely distributed prismatic particles, with the coexistence of two distinct phases. Meanwhile, cryogenic treatment, another thermal processing technique demonstrating remarkable efficacy in ferrous metallurgy, has recently garnered growing attention within Mg alloy research. Previous studies have demonstrated the beneficial effects of deep cryogenic treatment on magnesium alloy properties [10]. According to Jia et al. [11], the ultimate tensile strength (UTS) and yield strength (YS) of extruded Mg-9Gd-4Y-2Zn-0.5Zr alloy showed more significant enhancement when processed with deep cryogenic treatment compared to conventional methods. This improvement was attributed to the increased dislocation density induced by cryogenic conditions. Similarly, Hu et al. [12] observed that deep cryogenic treatment applied to rolled ZK60 alloy sheets promoted the formation of ultra-fine zinc-rich precipitates, while simultaneously creating elevated internal stress levels within the material. While substantial research efforts have been dedicated to elucidating the individual influence mechanisms of these two thermal processing approaches, the synergistic effects arising from their combined application remain insufficiently explored, thus this study is of great significance.

This paper systematically investigates the synergistic effects of aging treatments at varying temperatures and cryogenic treatment under identical conditions on the mechanical properties and microstructure evolution of extruded Mg-8Gd-3Y-0.4Zr alloys. Tensile testing was employed to evaluate mechanical properties, while advanced microstructure characterization techniques were utilized to elucidate the underlying mechanisms of microstructure transformation during aging and cryogenic processing. The research aims to uncover the interplay between thermal processing parameters and alloy strengthening mechanisms, thereby establishing a novel methodology for fabricating high-performance Mg alloys through optimized hybrid heat treatment.

## 2. Experimental Procedure

The as-cast Mg-8wt% Gd-3wt% Y-0.4wt% Zr rare-earth Mg alloy was utilized in this study. The bulk ingots of Mg-8Gd-3Y-0.4Zr alloy were prepared by direct chill casting with a casting speed of 100 mm/min, a melt temperature of 700 °C, and a water flow rate of 25 L/min, followed by homogenization at 500 °C for 9 h. The size of the ingot was 150 mm × 100 mm × 80 mm. The Mg-Gd-Y-Zr Mg alloy was sectioned into cylindrical billets with a diameter of 35 mm using electrical discharge wire cutting machine. These billets were subsequently extruded at a temperature of 420 °C and an extrusion speed of 0.5 mm/s with a force of 35 t, producing rods with a diameter of 8 mm, which were then air-cooled to room temperature. Based on the previous literature, these relatively appropriate extrusion parameters were selected and a 100 mm section was cut from the rod material [13]. Six groups of as-extruded samples were selected for further treatment and every group includes two samples: One group underwent isothermal holding at 210 °C for 20 h in a resistance heating furnace (A210). The remaining five groups were subjected to isothermal holding at 50, 90, 130, 170, and 210 °C for 20 h, respectively, followed immediately by cryogenic treatment via immersion in liquid nitrogen for 1 h (A50-CT, A90-CT, A130-CT, A170-CT, A210-CT).

The hardness of all samples was systematically tested at ten points from the edge to the center using a 200 HV-5 microhardness tester (Dong hua, Weifang, China) with a diamond square pyramid indenter, a load of 4.9 N and a dwell time of 15 s. To establish correlations between mechanical performance and microstructure evolution, many microstructure characterizations were implemented. OM involved sequential grinding, polishing and etching (the solution used for etching consists of 2.5 g picric acid, 2.5 mL acetic acid, 50 mL alcohol and 50 mL water) of specimens prior to observation under an MR500 inverted microscope (Aoka, Wuzhou, China). EBSD studies utilized an FEL Nova NanoSEM 450 system (FEI, Hillsboro, OR, USA) integrated with HKL Channel 5 software, operating at a step size of 0.75 μm and an accelerating voltage of 20 kV. For TEM, specimens were precision-sectioned to 5 × 5 mm dimensions via wire-electrical discharge machining, mechanically thinned, and final-polished using ion milling at 173 K. High-resolution imaging was subsequently conducted on an FEI Talos F200X TEM (ThermoFisher, Hilsboro, CA, USA) at 200 kV. Residual 20 mm long specimens were retained for supplementary microstructure investigations.

## 3. Results and Discussion

### 3.1. Microstructure Analysis

**Figure 1:**
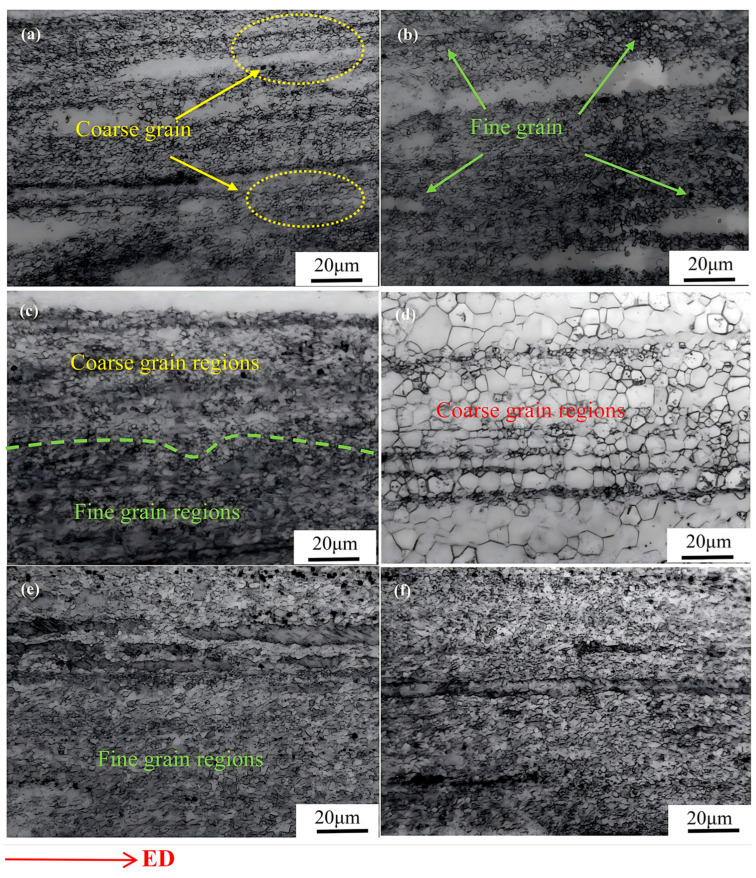
Optical microstructures of Mg-8Gd-3Y-0.4Zr alloy samples: (**a**) A50-CT, (**b**) A90-CT, (**c**) A130-CT, (**d**) A170-CT, (**e**) A210-CT, (**f**) A210.

The OM images of Mg-8Gd-3Y-0.4Zr alloy samples are shown in Figure 1. As shown in Figure 1, the grain size of Mg alloy samples was significantly refined after extrusion [14]. The grains in Mg alloy samples exhibit a streamlined uniform arrangement along the extrusion direction, yet the overall grain size distribution appears heterogeneous, with localized regions containing coarser grain structures. This phenomenon may be attributed to non-uniform stress distribution during the extrusion process, leading to different deformation degrees within the material. From Figure 1a–c, it is evident that when aging temperatures range between 50 °C and 130 °C, no significant changes in grain size were observed. However, the grain distribution becomes more homogeneous with increasing temperature. Figure 1d distinctly demonstrates substantial grain coarsening when aged at 170 °C, where most microstructure constituents transform into enlarged grains. This growth behavior results from enhanced energy supply at elevated temperatures, facilitating grain nucleation and subsequent growth during the aging process [15]. Figure 1e,f reveal that specimens subjected to deep cryogenic treatment after aging at 210 °C for 20 h exhibit smaller average grain sizes compared to those receiving solely aging treatment. This refinement mechanism stems from rapid lattice contraction induced by the abrupt temperature drop during cryogenic treatment, which effectively inhibits grain growth [12]. The experimental findings indicate aging treatment exert considerable influence on the hardness of Mg alloys. Compared with previous studies [16], the combined cryogenic and aging treatment developed in this work achieves notably superior grain refinement in Mg alloy samples.

**Figure 2:**
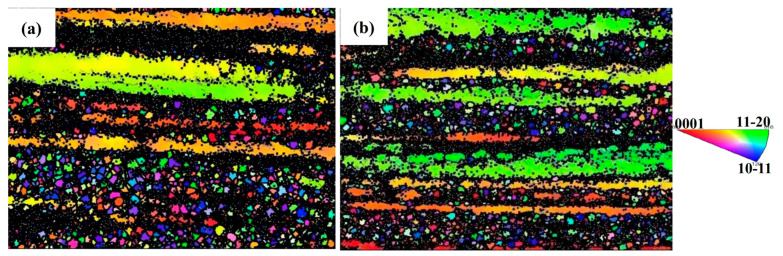
EBSD maps of Mg-8Gd-3Y-0.4Zr alloy samples: (**a**) A210-CT, (**b**) A210.

Figure 2 presents the EBSD maps of A210-CT and A210 samples. The dark regions represent areas where grain information could not be resolved. Gray lines denote low-angle grain boundaries (LAGBs) with misorientation angles ranging from 2° to 15°, while black lines correspond to high-angle grain boundaries (HAGBs) exhibiting misorientation angles exceeding 15°. As evidenced by the coloration of elongated grains, the rapid cooling during cryogenic treatment induces lattice contraction, generating additional internal stresses that consequently modify grain orientations [17]. Furthermore, comparative analysis reveals that the A210-CT specimen contains fewer and smaller elongated grains compared to the A210 specimen. This refinement phenomenon can be attributed to the inherent thermal contraction characteristics of metallic materials, where the temperature reduction during cryogenic processing effectively suppresses grain growth and promotes grain refinement [18]. The Hall–Petch relationship establishes that grain refinement substantially contributes to strength enhancement.

*σ_s_* = *σ*_0_ + *Kd*^−1/2^ (1)

where *σ_s_* is the yield stress, *σ*_0_ is the friction stress, *K* is the stress concentration factor, and *d* is the average grain size. Mg alloys exhibit a high Taylor factor, which amplifies the strengthening efficacy of grain refinement compared to other metal materials [19]. During extrusion, equiaxed grains are elongated along the deformation direction under shear or uniaxial stress. Complete dynamic recrystallization (DRX) during deformation facilitates the formation of equiaxed grains, while incomplete recrystallization preserves the elongated morphology of original grains. The regulatory effects of distinct DRX mechanisms on grain morphology—such as continuous dynamic recrystallization, discontinuous dynamic recrystallization, and twinning-induced dynamic recrystallization (CDRX/DDRX/TDRX)—vary with temperature, where elevated temperatures may preferentially facilitate equiaxed grain formation. Texture evolution or second-phase precipitation during deformation may inhibit recrystallization, resulting in elongated grains within non-recrystallized regions. These elongated grains likely originate from the aforementioned mechanisms [20,21]. Elongated grains improve material strength through multiple synergistic effects: the increased grain boundary area impedes dislocation motion and elevates dislocation density, amplifying dislocation strengthening, as quantified by Equation (2) [22].

(2) $$\sigma_{GB}=\sigma_{HAB}+\sigma_{LAB}=k_{y}{\left(d_{HAB}\right)\;}^{-1/2}+M\alpha G\;{\left(1.5bS_{V}\;\theta_{ave}^{LAB}\left(1-f_{HAB}\right)\right)}^{1/2}$$

where *k*_y_ is the Hall–Petch constant. *d_HAB_* is the size of grains surrounded by HAGB, $\theta_{ave}^{LAB}$ is the average misorientation angle of LAGBs and *f_HAB_* is the fraction of HAGBs. *S_V_* is the total area of GBs per unit volume, *M* is the Taylor factor, *α* is a constant, *G* is the shear modulus, and *b* is the magnitude of the Burgers vector. Simultaneously, pronounced grain orientation differences between elongated grains and adjacent fine-grained regions generate mutual grain boundary pinning effects. Furthermore, the constrained deformation space within elongated grains necessitates higher applied stresses to accommodate plastic deformation, collectively enhancing strength through these orientation and geometrically necessary dislocation (GND) interactions.

### 3.2. TEM Analysis

**Figure 3:**
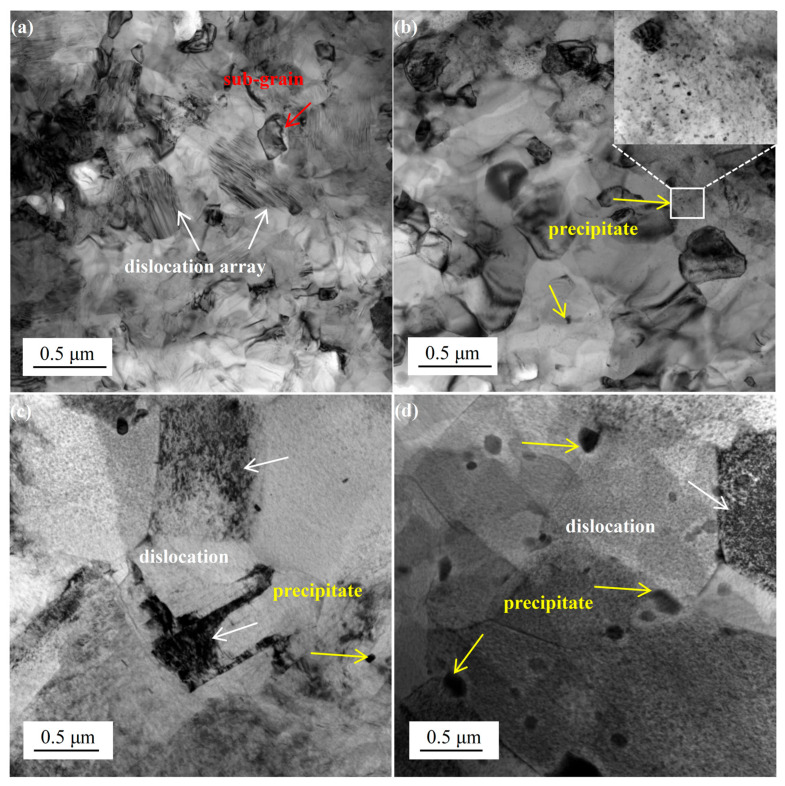
The TEM images of Mg-8Gd-3Y-0.4Zr alloy samples: (**a**,**b**) A130-CT, (**c**,**d**) A210-CT.

Figure 3 presents TEM images of A130-CT and A210-CT specimens. During the aging and deep cryogenic treatment processes, high-stress regions existing in grain interiors and grain boundaries of the microstructure provide sufficient energy for subsequent massive dislocation generation. Dislocation arrays and subgrain structures formed by the aggregation of numerous dislocations can be clearly observed in Figure 3a. Subgrains are formed in metallic materials through plastic deformation, where a large number of dislocations continuously accumulate and tangle to form dislocation walls. These dislocation walls further absorb dislocations and undergo rearrangement, thereby demarcating low dislocation density regions to establish subgrains. Subgrain boundaries still maintain the capability to hinder dislocation movement [23]. Considering the thermal expansion/contraction characteristics of metals, deep cryogenic treatment induces overall contraction of the alloy, leading to corresponding increases in internal dislocations and subsequent dislocation accumulation, ultimately forming high density dislocations. Figure 3b similarly reveals high-density dislocations but exhibits minimal second-phase particles, which are predominantly located within grains with notably small dimensions. In Figure 3c,d, although dislocation density shows some reduction, the population of second-phase particles increases significantly. These second-phase particles demonstrate limited distribution within grains, predominantly aligning along grain boundaries with apparent size enlargement. This indicates that increasing aging temperature may reduce dislocation density to some extent while promoting the precipitation and growth of second phase particles. Larger second phase particles can more effectively impede dislocation motion through dislocation strengthening mechanism. According to the Orowan mechanism, the strengthening effect of second phase particles can be described by Equations (3) and (4) [24].

(3) $$\sigma_{\mathrm{Orowan}}=M\frac{0.4G_{m}b}{\pi \lambda \left(\sqrt{1-\nu }\right)}\ln\left(\frac{r}{b}\right)$$

(4) $$\lambda =d\left(\sqrt{\frac{\pi }{4V_{p}}}-1\right)$$

Here, *M*, *r*, *ν*, *d* and *Vp* are the average orientation factor, the dispersoid radius of the precipitates, the Poisson’s ratio, the mean particle diameter and the volume fraction of particles, respectively. However, oversized second-phase particles may induce stress concentration, leading to localized hardness enhancement, resultant hardness inhomogeneity, and overall plasticity reduction. Based on this analysis, the A130-CT specimen primarily relies on high density dislocations for performance enhancement, whereas the A210-CT specimen demonstrates higher strength contribution from second-phase particles.

**Figure 4:**
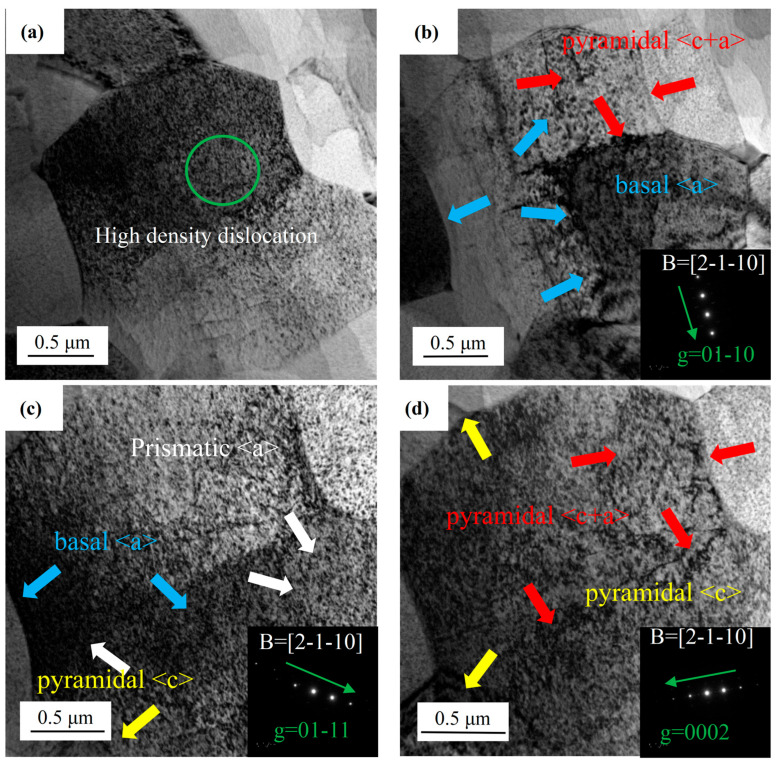
Two-beam bright-field TEM micrographs of A210-CT sample: (**a**) high-density dislocation, (**b**) g = 01-10, (**c**) g = 01-11, (**d**) g = 0002.

**Figure 5:**
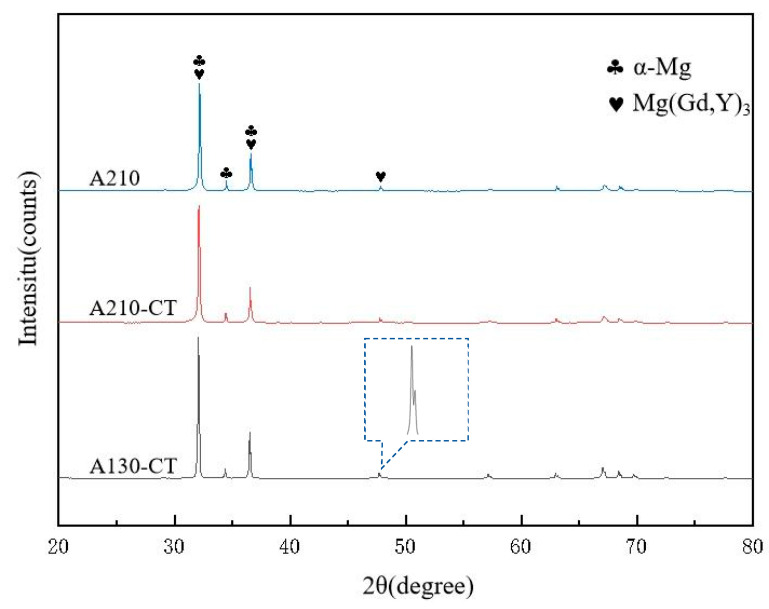
XRD diffraction pattern of Mg-8Gd-3Y-0.4Zr alloy samples.

To further characterize dislocation types under different diffraction vectors (g), two-beam bright-field TEM micrographs of A210-CT are presented in Figure 4. Through calibration analyses [25,26], the diffraction direction was determined as B = [2-1-10]. The vector direction from dark to bright light spots corresponds to the (**g**) vector orientation, with the brightest spot’s crystal plane index representing the (**g**) vector magnitude. Dislocation characterization was performed by evaluating **g**·**b** values (where **b** denotes the Burgers vector), guided by the **g**·**b** = 0 invisibility criterion. Under the two-beam diffraction condition with **g** = (01-10), pyramidal <c> dislocations become invisible, while <a>-type dislocations and pyramidal <c+a> dislocations remain observable. Conversely, <a>-type dislocations are not detectable under **g** = (0002) diffraction. All dislocation types (<a>, <c>, and <c+a>) exhibit visibility in the **g** = (01-11) diffraction condition. By systematically comparing the dislocation contrasts across **g** = (01-10), **g** = (0002), and **g** = (01-11) diffraction modes, the dislocation types were identified as illustrated in Figure 5 (annotated with arrows and dashed boxes). This multi-vector analysis enables precise discrimination of dislocation configurations based on their distinct visibility responses to specific diffraction vectors.

Figure 5 presents the XRD patterns of three magnesium alloy samples (A210, A210-CT, and A130-CT). All samples exhibited prominent diffraction peaks at characteristic α-Mg phase positions (~34, ~36, ~47.8°, etc.), confirming the magnesium matrix as the primary phase. The diffraction peaks corresponding to the Mg(Gd,Y)_3_ phase (observed in the ~30–40° range and near ~50°) demonstrated enhanced intensities in both cryogenically treated samples (A210-CT and A130-CT), with the A130-CT specimen showing particularly pronounced peak intensities (~56,000). This indicates that cryogenic treatment effectively promotes the precipitation of this rare-earth-containing phase. Notably, the Mg(Gd,Y)_3_ peaks in A130-CT displayed narrower full width at half maximum coupled with higher intensities, suggesting that elevated aging temperatures might accelerate the growth and coarsening of precipitated phases. This observation aligns with previous TEM findings showing increased second phase particle sizes. The A210 sample exhibited peak broadening in specific angular ranges (like, ~45–60°), potentially indicative of localized microstrain in non-cryotreated specimens. In contrast, the sharper peak profiles observed in CT-processed samples imply that cryogenic treatment can facilitates internal stress relief and residual stress mitigation. On the other hand, due to the quantity and size of the second phase, as well as the selected area of the test sample and other reasons, there will be errors in the test results, leading to the lack of calibration for some of the second phase [27].

**Figure 6:**
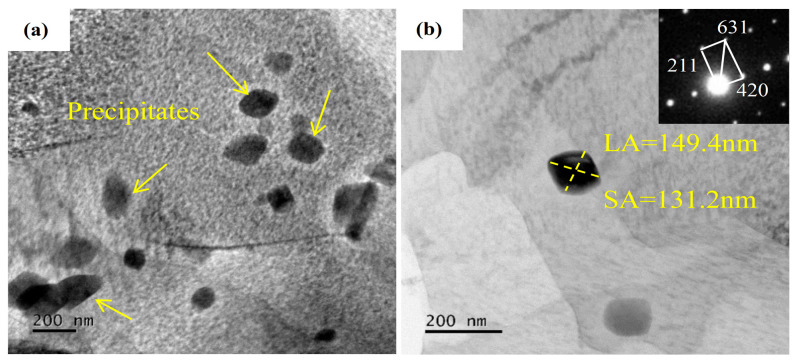
The TEM images of precipitates of A210-CT sample. (**a**) precipitates, (**b**) precipitate size and identification.

Figure 6 presents the morphological characteristics and phase identification results of second phases in the A210-CT sample. The second phases exhibit irregular-shaped morphology with dense distribution and relatively large dimensions, predominantly exceeding 100 nm. Although these coarse and densely distributed second phases may enhance strength and hardness, they tend to induce stress concentration, potentially compromising ductility and causing inhomogeneous hardness distribution. Figure 6b displays the second-phase morphology and corresponding diffraction patterns in the A210-CT sample. The identified second phase corresponds to Mg(Gd,Y)_3_, with measured cross-sectional dimensions of 149.4 nm (long-axis length, L) and 131.2 nm (short-axis length, S) for the precipitates. The Mg(Gd,Y)_3_ phase serves as an efficient strengthening component distributed in the magnesium matrix, leading to significant enhancement of the alloy’s mechanical properties. Due to the high thermal stability exhibited by Gd and Y alloying elements, the Mg(Gd,Y)_3_ phase maintains structural integrity under elevated temperature conditions, thereby contributing to the improvement of strength retention at high-temperature service environments [28,29].

### 3.3. Microhardness Analysis

**Figure 7:**
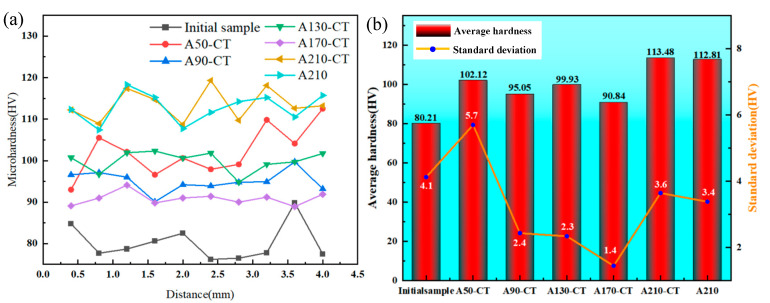
The images of the hardness experiment result: (**a**) the line graph of the hardness distribution and (**b**) the average hardness and the variance.

Figure 7 presents the hardness measurement results of magnesium alloy samples. The average hardness values for the Initial sample, A50-CT, A90-CT, A130-CT, A170-CT, A210-CT, and A210 samples were measured as 80.2, 102.1, 95.1, 99.3, 90.8, 113.5, and 112.8 HV, respectively. These results demonstrate that the aging–cryogenic combined treatment significantly enhances material hardness. Comparative analysis between the A210-CT and A210 samples reveals that aging treatment contributes more substantially to hardness improvement, while cryogenic treatment exhibits limited influence. Combined with previous microstructure characterization, this phenomenon may be attributed to cryogenic treatment’s dual effects of grain refinement (enhancing properties) and residual stress balancing, resulting in non-significant net hardness enhancement. Notably, the A210-CT sample exhibits superior average hardness compared to those treated at lower aging temperatures, indicating that higher aging temperatures within a specific range facilitate greater hardness improvement. TEM analysis suggests this enhancement originates from the precipitation and coarsening of second phases (Mg(Gd,Y)_3_) within the magnesium matrix during high-temperature aging. Equation (5) is the equation of variance which can be applied to express the level of uniformity of the value of the hardness distribution.

(5) $$\sigma_{v}=\frac{1}{n}\sum_{i=1}^{n}{\left(X_{i}-X_{avg}\right)}^{2}$$

where n = 10, σ_v_ is the variance, X_i_ is the hardness value of every test point and X_avg_ is the sample’s average hardness value. The standard deviations of hardness values for the initial sample and the A50-CT, A90-CT, A130-CT, A170-CT, A210-CT, and A210 samples were calculated as 4.1, 5.4, 2.3, 1.4, 3.6, and 3.4, respectively. The A210-CT and A210 samples show comparable hardness uniformity, while the A50-CT sample displays the poorest homogeneity and the A170-CT sample exhibits optimal uniformity. This trend suggests that insufficient aging temperatures may lead to non-uniform precipitation distribution of second phases, thereby degrading hardness uniformity. Moderate aging temperatures promote balanced precipitation behavior without excessive phase coarsening, effectively maintaining homogeneity [30]. However, excessively high aging temperatures induce precipitate coarsening and localized stress concentration, ultimately deteriorating hardness uniformity [31].

## 4. Conclusions

In this study, the influence of aging and cryogenic treatment on microstructure and hardness of extruded Mg-8Gd-3Y-0.4Zr alloy is investigated. The conclusions are as follows.

(1) The combined aging and cryogenic treatment enhances hardness, with A210-CT showing the highest average hardness (113.5 HV) while A170-CT exhibits optimal uniformity (variance: 1.7). A210 achieves comparable hardness (112.8 HV) to A210-CT.
(2) Higher aging temperatures promote hardness by facilitating second-phase precipitation at grain boundaries and interiors, though this reduces uniformity (A210-CT variance: 3.6).
(3) The cryogenic treatment exhibits limited effectiveness in enhancing hardness. While this process improves hardness through grain refinement, lattice contraction, inducing additional stresses, its concurrent reduction of residual stresses ultimately constrains hardness improvement.

## Data Availability

The original contributions presented in this study are included in the article. Further inquiries can be directed to the corresponding authors.
